# Childhood Physical Fitness as a Predictor of Cognition and Mental Health in Adolescence: The PANIC Study

**DOI:** 10.1007/s40279-024-02107-z

**Published:** 2024-09-10

**Authors:** Eero A. Haapala, Marja H. Leppänen, Hannamari Skog, David R. Lubans, Anna Viitasalo, Niina Lintu, Petri Jalanko, Sara Määttä, Timo A. Lakka

**Affiliations:** 1 Sports and Exercise Medicine, Faculty of Sport and Health Sciences, University of Jyväskylä, Jyväskylä, Finland; 2 Institute of Biomedicine, School of Medicine, University of Eastern Finland, Kuopio, Finland; 3 Centre for Active Living and Learning, College of Human and Social Futures, University of Newcastle, Callaghan, NSW Australia; 4 Hunter Medical Research Institute, New Lambton Heights, NSW Australia; 5 Helsinki Clinic for Sports and Exercise Medicine, Foundation for Sports and Exercise Medicine, Helsinki, Finland; 6 Department of Clinical Neurophysiology, Kuopio University Hospital, Kuopio, Finland; 7 Department of Clinical Physiology and Nuclear Medicine, Kuopio University Hospital, Kuopio, Finland; 8 Foundation for Research in Health Exercise and Nutrition, Kuopio Research Institute of Exercise Medicine, Kuopio, Finland

## Abstract

**Background:**

Cognitive and mental health problems are highly prevalent in adolescence. While higher levels of physical fitness may mitigate these problems, there is a lack of long-term follow-up studies on the associations of physical fitness from childhood with cognition and mental health in adolescence.

**Objective:**

We investigated the associations of physical fitness from childhood to adolescence over an 8-year follow-up with cognition and mental health in adolescence.

**Methods:**

The participants were 241 adolescents (112 girls), who were 6–9 years at baseline and 15–17 years at 8-year follow-up. Average and change scores for cardiorespiratory fitness (maximal power output [*W*_max_]; peak oxygen uptake [*V*O_2peak_]), motor fitness (10 × 5-m shuttle run), and muscular fitness (standing long jump; hand grip strength) were calculated. Global cognition score was computed from six individual cognitive tasks, and perceived stress and depressive symptoms were assessed at the 8-year follow-up. The data were analysed using linear regression models adjusted for age, sex, and parental education.

**Results:**

Average motor fitness was positively associated with global cognition score (standardised regression coefficient [*β*] − 0.164, 95% confidence interval [CI] − 0.318 to − 0.010) and inversely with perceived stress (*β* = 0.182, 95% CI 0.032–0.333) and depressive symptoms (*β* = 0.181, 95% CI 0.028–0.333). Average cardiorespiratory fitness was inversely associated with perceived stress (*W*_max_: *β* =  − 0.166, 95% CI − 0.296 to − 0.036; *V*O_2peak_: *β* =  − 0.149, 95% CI − 0.295 to − 0.002) and depressive symptoms (*W*_max_: *β* =  − 0.276, 95% CI − 0.405 to − 0.147; *V*O_2peak_: *β* =  − 0.247, 95% CI − 0.393 to − 0.102). A larger increase in cardiorespiratory fitness was associated with lower perceived stress (*W*_max_: *β* =  − 0.158, 95% CI − 0.312 to − 0.003; *V*O_2peak_: *β* =  − 0.220, 95% CI − 0.395 to − 0.044) and depressive symptoms (*W*_max_: *β* =  − 0.216, 95% CI − 0.371 to − 0.061; *V*O_2peak_: β =  − 0.257, 95% CI − 0.433 to − 0.080).

**Conclusions:**

Higher levels of motor fitness in childhood and adolescence were associated with better cognition in adolescence. Higher levels of and larger increases in cardiorespiratory fitness from childhood to adolescence were associated with better mental health in adolescence.

**Supplementary Information:**

The online version contains supplementary material available at 10.1007/s40279-024-02107-z.

## Key Points

**Table:** 

The results of our longitudinal study indicate that higher motor fitness in childhood and adolescence is associated with better cognition in adolescence.
Furthermore, we found that higher cardiorespiratory fitness in childhood and adolescence is associated with lower perceived stress and depressive symptoms in adolescence.
Our findings advocate for the investment in physical fitness from early life as a potential strategy for mitigating cognitive and mental health issues in adolescence.

## Introduction

Childhood and adolescence are critical periods for cognitive and mental health development [1, 2] due to remarkable changes in brain structures and functions as well as social and health-related behaviours during youth [3–5]. Deficits in cognition can lead to reduced educational and occupational status and poorer health in adulthood [6]. Moreover, the majority of mental illnesses emerge during adolescence and early adulthood [7]. Up to 25–30% of adolescents experience impaired mental health [8], and a third of adolescents have been estimated to be at risk of developing clinical depression [9]. Moreover, impaired mental health is also often associated with deteriorated cognition in youth [10]. Therefore, it is crucial to identify factors that promote cognition and mental health from childhood to adolescence. Such knowledge advances scientific research but also provides valuable insights for informing practical interventions and health policies.

While physical fitness has been identified as an important determinant of general health [11], today’s youth face challenges with low levels of physical fitness potentially compromising their cognition and elevating the risk of mental health problems [12]. Cardiorespiratory, motor, and muscular fitness have been positively associated with cognition in children and adolescents, but the majority of evidence is derived from cross-sectional studies [13–16]. Moreover, most studies have focused on the role of cardiorespiratory fitness, with few studies examining the role of motor and muscular fitness [13–15]. Furthermore, cross-sectional evidence suggests that cardiorespiratory fitness is inversely associated with depressive symptoms in children and adolescents [17]. Nevertheless, the few available studies suggest no association of motor or muscular fitness with depression [17]. In these studies, there is also considerable variation and weaknesses in adjustments for possible confounding factors, such as socioeconomic status and screen time, which could weaken the association between physical fitness and cognition [17, 18]. Moreover, evidence on the associations of cardiorespiratory fitness with cognition and mental health mainly stems from studies using measures of cardiorespiratory fitness from field tests, such as 20-m shuttle run test performance [17–19], but not more accurate measures of cardiorespiratory fitness, such as peak oxygen uptake (*V*O_2peak_) during a cycle ergometer or treadmill exercise test.

Participation in physical activity of sufficient volume and intensity to improve physical fitness has the potential to modify the structure, such as the hippocampal volume and white matter integrity, and function of several brain regions [18, 20]. In addition to the potential role of lifestyle factors affecting brain structures and functions, there are also remarkable differences in brain and cognitive development and the prevalence of mental health problems between girls and boys [3, 21]. Boys’ brains exhibit greater variability in several brain structures [21], and their frontal and parietal grey matter volumes peak approximately two years later than girls [3]. Additionally, depressive symptoms are more prevalent in girls than in boys [9]. Accordingly, some studies suggest sex-dimorphic associations of physical fitness with cognition in children and adolescents [17, 22, 23]. For example, we found that better motor performance predicted better cognition 2 years later in pre-pubertal boys but not in girls [22]. However, there is a lack of long-term follow-up studies providing evidence on sex differences in the associations of physical fitness from childhood with cognition and mental health in adolescence.

International scientific experts recently identified studies on longitudinal associations between physical fitness and health outcomes as the number one priority for research and surveillance on physical fitness among children and adolescents [24]. The development of physical fitness-induced cognitive and mental health alterations might require relatively long exposure to low or high levels of physical fitness, often commencing in childhood. We, therefore, examined the longitudinal associations of average physical fitness in childhood and adolescence and changes in physical fitness between childhood and adolescence with cognition and mental health in adolescence in a general population of children followed up for 8 years until adolescence. In secondary analyses, we also investigated whether sex modified these longitudinal associations.

## Methods

### Study Population and Study Design

The present data are from the Physical Activity and Nutrition in Children (PANIC) study, which was initially an 8-year physical activity and diet intervention study in a population sample of children from the city of Kuopio, Finland [25]. In the present analyses, the intervention and control groups were combined, treating the participants as a longitudinal cohort because the main outcomes were assessed only at the 8-year follow-up. The Research Ethics Committee of the Hospital District of Northern Savo, Kuopio, Finland, approved the study protocol in 2006 (Statement 69/2006). The parents or caregivers of the children gave their written informed consent, and the children provided their assent to participate. The PANIC study was carried out in accordance with the principles of the Declaration of Helsinki, as revised in 2008.

Altogether, 736 children aged 6–9 years from primary schools in Kuopio were invited to participate in the baseline examination in 2007–2009. After the baseline assessments, the participants were followed up for 2 years (aged 8–11 years) and 8 years (aged 15–17 years) after baseline. Inclusion criteria for participation were apparently healthy boy or girl, 6–9 years of age at baseline, living in the city of Kuopio, Finland. The exclusion criteria included physical disabilities, psychosocial and neurodevelopmental disorders, as well as mental health disorders, which could hamper participation in the intervention.

The adolescents who did not participate in the 8-year follow-up did not differ in sex, age, pubertal stage, height, weight, and body mass index standard deviation score (BMI-SDS) at baseline from those who participated in the 8-year follow-up (all *p* > 0.10 for the difference). Those who did not participate in the 8-year follow-up assessments had higher body fat percentage (BF%) at baseline (mean difference 2.4, 95% confidence interval [CI] 0.8–4.0) and were more likely to be from families without parents with university-level education at baseline (27% vs 43%; *p* < 0.001) than those who participated in the 8-year follow-up.

### Assessments

Cardiorespiratory, motor, and muscular fitness were assessed at baseline and at the 2-year and 8-year follow-up. Cognition and mental health were assessed at the 8-year follow-up. The assessments were carried out in the same way at all three time points over 1 month in the following order: (1) body height and weight, pubertal status, muscular fitness by hand grip strength, and cardiorespiratory fitness, (2) body composition by DXA device and cognition by the Raven’s matrices test, and (3) muscular fitness by the standing long jump test, and motor fitness by the 10 × 5-m shuttle run test. Psychomotor function, attention, reaction time in the working memory task, working memory accuracy, and visual memory and learning were assessed at the second visit of the 8-year follow-up. Most assessments were performed at the Institute of Biomedicine, University of Eastern Finland. Body composition by DXA was assessed at the Department of Clinical Physiology and Nuclear Medicine, Kuopio University Hospital.

#### Assessment of Cognition at Baseline and 8-Year Follow-Up

A global cognition score, which is based on the results of six individual cognitive tasks assessed by the Raven’s Standard Progressive Matrices (SPM) [26, 27] and a computerised CogState test battery (CogState Ltd, Melbourne, Australia) [28] was used as a measure of cognition at 8-year follow-up (see Online Resource S1 in the electronic supplementary material [ESM] for details). In short, the paper–pencil version of the Raven’s SPM was used to assess non-verbal reasoning skills and the CogState test battery to assess psychomotor function (the detection task), attention (the identification task), reaction time in working memory task (the one-back task), working memory accuracy (the two-back task), and visual memory and learning (the continuous paired associate learning task). Following the approach of the Finnish Geriatric Intervention Study to Prevent Cognitive Impairment and Disability (FINGER) study [29], a global cognition score was calculated by summing the z-scores across the Raven’s SPM and all CogState tests as *Z*_Raven’s SPM_ + *Z*_psychomotor function_ + *Z*_attention_ + *Z*_reaction time in working memory_ + *Z*_working memory accuracy_ + *Z*_paired associate learning_. In adults, composite cognition scores have been found to be a more sensitive measure to detect mild cognitive impairment than individual components of cognition [30]. For the tests for which a lower score indicated better performance, the score was reversed (multiplied by − 1) so that all cognition variables were in a uniform direction. A higher score indicated better global cognition. Childhood cognition at baseline as a confounding factor was assessed using the Raven’s Coloured Progressive Matrices [27, 31].

#### Assessment of Mental Health at 8-Year Follow-Up

Perceived stress was assessed by the Cohen’s Perceived Stress Scale [32]. The Finnish version of the Perceived Stress Scale contains 10 questions, each scoring from 0 to 4, and the scale thus ranging between 0 and 40 points. The Cohen’s Perceived Stress Scale is a valid measure for detecting perceived stress in youth [33]. Depressive symptoms were assessed by the Beck’s Depression Inventory that measures characteristic attitudes and symptoms of depression and contains 21 questions, each scoring from 0 to 3, with the scale thus ranging between 0 and 63 points [34]. The Beck’s Depression Inventory is a suitable tool for assessing depressive symptoms in adolescents [35, 36]. A higher perceived stress score indicates higher levels of perceived stress, and a higher depressive symptoms score indicates higher levels of depressive symptoms.

#### Assessment of Cardiorespiratory Fitness at Baseline, 2-Year Follow-Up, and 8-Year Follow-Up

Cardiorespiratory fitness was assessed at baseline, 2-year follow-up, and 8-year follow-up by a maximal exercise test using an electromagnetically braked Ergoselect 200 K^®^ cycle ergometer coupled with a paediatric saddle module (Ergoline, Bitz, Germany) [37]. The exercise test protocol included a 2.5-min anticipatory period with the child sitting on the ergometer; a 3-min warm-up period with a workload of 5 watts; a 1-min steady-state period with a workload of 20 watts; an exercise period with an increase in the workload of 1 W per 6 s until exhaustion; and a 4-min recovery period with a workload of 5 watts. The children were asked to keep the cadence stable and within 70–80 revolutions per minute. Exhaustion was defined as inability to maintain the cadence above 65 revolutions per minute regardless of vigorous verbal exhortation. The exercise tests were considered maximal by an experienced physician supervising the tests if objective and subjective criteria (maximal heart rate > 85% of predicted, sweating, flushing, inability to continue exercise test regardless of strong verbal encouragement) indicated maximal effort and maximal cardiovascular capacity [37]. Only those tests considered maximal were included in the analyses (486 tests at baseline, 426 tests at 2-year follow-up, and 239 tests at 8-year follow-up). At 2-year and 8-year follow-up, $\dot{V}$O_2peak_ (mL × min^−1^) was additionally assessed during an incremental exercise test by the Oxycon Pro^®^ respiratory gas analyser (Jaeger, Hoechberg, Germany). Therefore, we used maximal power output expressed as Watts (*W*_max_) as a measure of cardiorespiratory fitness at all time points and $\dot{V}$O_2peak_ at 2-year and 8-year time points. *W*_max_ and $\dot{V}$O_2peak_ were normalised for kilogram of lean body mass (LBM) [38].

#### Assessment of Motor Fitness at Baseline, 2-Year Follow-Up, and 8-Year Follow-Up

Motor fitness in terms of speed and agility was assessed by the 10 × 5-m shuttle run test [39]. Average motor fitness across three time points had a strong positive correlation (*r* = 0.755, *p* < 0.001) with average lower limb power assessed by the standing long jump test across all three time points in the present study sample. The children were asked to run 5 m from a starting line to another line as fast as possible, turn on the line, run back to the starting line, and continue until five shuttles were completed. The test score was the running time in seconds, with a longer time indicating a poorer performance. The test was performed once. The 10 × 5-m shuttle run test has been found to be reliable with an intraclass correlation of 0.69 between the measurements taken one week apart [40], and the 4 × 10-m speed and agility shuttle run test has been reported to have moderate to good reproducibility with a 0.1-s inter-trial difference [41].

#### Assessment of Muscular Fitness at Baseline, 2-Year Follow-Up, and 8-Year Follow-Up

Lower limb power was assessed by the standing long jump test [39]. The children were asked to stand with their feet together, jump as far as possible, and land on both feet. The test score was the longest jump of three attempts in centimetres. Hand grip strength was assessed by the Martin vigorimeter (Martin, Tuttlingen, Germany). The children were asked to keep the elbow close to the body with the arm flexed at 90° and to squeeze a rubber bulb as hard as possible with the dominant hand. The test was performed three times. The best result was used in the analyses. Hand grip strength was expressed in kilopascals (kPa) and scaled by kilogram of LBM. Both of these tests have been found to have an acceptable reproducibility in youth [11, 41].

#### Average Physical Fitness Scores and Changes in Measures of Physical Fitness

Average physical fitness scores across the 8-year follow-up for each measure of physical fitness were computed by summing their *z*-scores at baseline, 2-year follow-up, and 8-year follow-up and dividing the sum by three [42]. The average $\dot{V}$O_2peak_ score was computed by summing *z*-scores for $\dot{V}$O_2peak_ at 2-year and 8-year follow-up and dividing the sum by 2. Changes in measures of physical fitness from baseline to 8-year follow-up were computed by subtracting their baseline values from their 8-year follow-up values. A change in $\dot{V}$O_2peak_ was computed, subtracting their 2-year follow-up values from their 8-year follow-up values.

#### Assessment of Body Height, Weight, and Composition at 8-Year Follow-Up

Body weight was measured twice with the participant having fasted for 12 h, emptied the bladder, and standing in light underwear using a weight scale integrated into a calibrated InBody^®^ 720 bioelectrical impedance device (Biospace, Seoul, South Korea) to an accuracy of 0.1 kg. The mean of these two values was used in the analyses. Body height was measured three times with the participant standing in the Frankfurt plane without shoes using a wall-mounted stadiometer to an accuracy of 0.1 cm. The mean of the nearest two values was used in the analyses. BMI was calculated by dividing weight (kg) by height (m) squared. BMI-SDS was calculated based on Finnish reference data [43]. The prevalence of overweight and obesity was defined using the cut-off values provided by Cole and Lobstein [44]. Body fat mass, BF%, and LBM were measured by the Lunar^®^ DXA device (GE Medical Systems, Madison, WI, USA) using a standardised protocol [45].

#### Other Assessments

A research physician assessed pubertal status at the 8-year follow-up using a 5-stage scale described by Marshall and Tanner [46, 47]. Testicular volume assessed by an orchidometer was used as a measure of pubertal status in boys and breast development in girls. The parents were asked to report in a questionnaire their completed or ongoing educational degrees categorised as vocational school or less, polytechnic, and university. The degree of the more educated parent was used in the analyses. Total physical activity and total screen time at 8-year follow-up were assessed by the PANIC Physical Activity Questionnaire [48]. Total screen time included the use of different screens, such as TV, computer, game consoles, tablets, and mobile phones. Physical activity questionnaires with a similar structure to the PANIC Physical Activity Questionnaire, such as the Youth Physical Activity questionnaire, have shown good short-term repeatability over 4 days with an intraclass correlation of 0.86–0.92 [49].

### Statistical Methods

Statistical analyses were performed using the jamovi software, version 2.2.5.0 (The jamovi project 2021). The characteristics of girls and boys were compared using the Welch’s test for normally distributed continuous variables, the Mann–Whitney’s *U*-test for continuous variables with skewed distributions, and the *χ*^2^-test for categorical variables.

The associations of the average physical fitness scores and changes in measures of physical fitness over 8 years with global cognition score, perceived stress, and depressive symptoms at 8-year follow-up were investigated using linear regression analyses adjusted for age, sex, and parental education at 8-year follow-up. The data on the associations of the changes in physical fitness measures with the global cognition score, perceived stress, and depressive symptoms were also adjusted for the baseline value of each measure of physical fitness. These data were further adjusted for pubertal status, BF%, physical activity, or screen time at 8-year follow-up. These covariates were entered separately into the models. We also examined whether the intervention had an effect on the associations by incorporating the study group variable (intervention vs control group) into the models. The study group had no effect on the associations and was not considered further. The data regarding the global cognition score were additionally adjusted for the Raven’s score at baseline. The data on the associations between physical fitness and cognitive functions were also adjusted for perceived stress and depressive symptoms, whereas the corresponding data regarding perceived stress and depressive symptoms were adjusted for the global cognition score. These covariates were entered separately into the models. To study the modifying effect of sex on the associations of indices of physical fitness with global cognition score, perceived stress, and depressive symptoms, we included a sex × physical fitness interaction term in the models. The associations of measures of physical fitness at different time points with individual measures of cognition and mental health at 8-year follow-up are presented in the Online Resource S2 and Online Resource Tables S1–S3 (see ESM). The data are presented as standardised regression coefficients (*β*) and their 95% confidence intervals (95% CI). We considered *β* of 0.10–0.29, 0.30–0.49, and ≥ 0.50 to describe small, moderate, and strong magnitude of the associations, respectively [50].

## Results

### Participants

A total of 512 children, representing 70% of those invited, completed the baseline examinations. Six children were excluded from the study at baseline because of physical disabilities that could hamper participation in the intervention or no time or motivation to attend in the study. We also excluded data from two children whose parents or caregivers later withdrew their permission to use these data in the study. The participants did not differ in sex distribution, age, or BMI-SDS from all children who started the first grade in 2007–2009 based on data from the standard school health examinations performed for all Finnish children before the first grade (data not shown). Data on variables used in the analyses were available for 241–262 participants at baseline, 215–256 participants at 2-year follow-up, and for 213–241 participants at 8-year follow-up. The maximal number of participants for the analyses of physical fitness with cognition and mental health was 241. None of the participants included in the present analyses had attention-deficit hyperactivity disorder or other neurodevelopmental disorders.

### Characteristics of the Participants

The characteristics of participants at 8-year follow-up are presented in Table 1. Girls had a lower motor fitness and standing long jump performance but higher hand grip strength than boys. Girls also had higher perceived stress and depressive symptoms scores than boys. There was no difference in the global cognition score between girls and boys.

**Table 1:** Characteristics of participants at 8-year follow-up

	All (*N* = 241)	Girls (*N* = 112)	Boys (*N* = 129)	*p* value
Age (y)	15.8 (0.4)	15.7 (0.4)	15.8 (0.7)	0.540
Height (cm)	171.5 (8.6)	165.0 (5.9)	177.0 (7.4)	** < 0.001**
Body weight (kg)	62.0 (8.6)	57.9 (9.1)	65.5 (14.0)	** < 0.001**
Body mass index standard deviation score	− 0.05 (1.0)	0.05 (0.9)	− 0.13 (1.1)	0.170
Prevalence of overweight/obesity (%)	12.9	11.6	14.0	
Body fat percentage (%)	23.0 (13.3 to 29.5)	28.8 (23.9 to 32.2)	16.9 (10.5 to 21.1)	** < 0.001**
Pubertal status (range 1–5) (%)				**0.002**
3	8	4	12	
4	58	54	62	
5	33	42	25	
Physical activity (min/wk)	129 (78.0 to 189.0)	106 (73.2 to 157)	154 (81.7 to 221)	**0.002**
Screen time (min/wk)	299 (223 to 407)	265 (201 to 400)	341 (248 to 420)	**0.003**
Parental education (%)				**0.011**
Vocation school or less	14	14	13	
Polytechnic	40	48	32	
University	47	38	55	
Cardiorespiratory fitness				
*W*_max_/kg of LBM (*n* = 231)	4.4 (0.6)	4.4 (0.5)	4.4 (0.6)	0.273
*V*O_2peak_/kg of LBM (*n* = 210)	61.8 (6.3)	61.0 (5.6)	62.4 (6.8)	0.114
Motor fitness				
10 × 5-m shuttle run test (s) (*n* = 234)	20.3 (1.7)	21.6 (1.7)	19.9 (2.0)	** < 0.001**
Muscular fitness				
Standing long jump (cm) (*n* = 233)	192 (31.8)	172 (22.9)	209 (28.6)	** < 0.001**
Handgrip strength (kPa/kg of LBM)	2.59 (0.56)	2.8 (0.5)	2.4 (0.5)	** < 0.001**
Global cognition score (*n* = 239)	− 0.00 (0.59)	0.02 (0.6)	− 0.01 (0.6)	0.058
Perceived stress score	12.8 (5.9)	14.8 (5.7)	11.0 (10.0)	** < 0.001**
Depressive symptoms score (*n* = 239)	1.0 (0.0 to 4.0)	2.0 (0.0 to 6.5)	1.0 (0.0 to 2.3)	** < 0.001**

The data are means (standard deviations) or medians (interquartile ranges)

*p* value for the differences between girls and boys are from the Welch’s test, the Mann–Whitney *U*-test, or the *χ*^2^ test

The percentages describing pubertal status and parental education were rounded, and therefore they do not equal 100%

Bolded *p* values denote statistically significant differences between girls and boys

*kPa* kilopascal, *LBM* lean body mass, *VO*_*2peak*_ peak oxygen uptake, *W*_*max*_ maximal workload

### Associations of Average Physical Fitness and Changes in Physical Fitness Over 8 Years with Cognition at 8-Year-Follow-Up

A higher average motor fitness was associated with a higher global cognition score at the 8-year follow-up after adjustment for age, sex, and parental education (Table 2). The magnitude of this association remained similar after further adjustment for pubertal status, BF%, physical activity, or screen time at the 8-year follow-up or the Raven’s Coloured Progressive Matrices score at baseline, which were entered separately into the models. Other measures of average physical fitness or changes in the measures of physical fitness from baseline to 8-year follow-up were not associated with cognition.

**Table 2:** Associations of average physical fitness and changes in physical fitness over 8 years with cognition and mental health at 8-year follow-up

	*N*	Global cognition score	*N*	Perceived stress score	*N*	Depressive symptoms score
Average physical fitness						
Cardiorespiratory fitness (*W*_max_/kg of LBM)	217	0.047 (− 0.090 to 0.183)	218	** − 0.166 (− 0.296 to − 0.036)**	216	** − 0.276 (− 0.405 to − 0.147)**
Cardiorespiratory fitness (*V*O_2peak_ /kg of LBM)	183	0.051 (− 0.100 to 0.202)	184	** − 0.149 (− 0.295 to − 0.002)**	183	** − 0.247 (− 0.393 to − 0.102)**
Motor fitness (10 × 5-m shuttle run test, s)	189	** − 0.164 (− 0.318 to − 0.010)**	193	**0.182 (0.032 to 0.333)**	189	**0.181 (0.028 to 0.333)**
Muscular fitness (standing long jump, cm)	191	0.097 (− 0.072 to 0.267)	191	− 0.144 (− 0.301 to 0.022)	191	− 0.162 (− 0.332 to 0.007)
Muscular fitness (handgrip strength, kPa/kg of LBM)	227	− 0.038 (− 0.170 to 0.095)	229	− 0.049 (− 0.177 to 0.079)	227	− 0.040 (− 0.172 to 0.091)
Change in physical fitness						
Cardiorespiratory fitness (*W*_max_/kg of LBM)	224	0.079 (− 0.085 to 0.243)	226	** − 0.158 (− 0.312 to − 0.003)**	224	** − 0.216 (− 0.371 to − 0.061)**
Cardiorespiratory fitness (*V*O_2peak_ /kg of LBM)	183	0.116 (− 0.066 to 0.298)	184	** − 0.220 (− 0.395 to − 0.044)**	183	** − 0.257 (− 0.433 to − 0.080)** 0.091 (− 0.099 to 0.280)
Motor fitness (10 × 5-m shuttle run test, s)	222	− 0.156 (− 0.339 to 0.026)	224	0.114 (− 0.066 to 0.293)	222	
Muscular fitness (standing long jump, cm)	222	− 0.018 (− 0.18 to 0.144)	224	− 0.093 (− 0.232 to 0.046)	222	− 0.008 (− 0.150 to 0.133)
Muscular fitness (handgrip strength, kPa/kg of LBM)	235	− 0.068 (− 0.217 to 0.081)	237	− 0.036 (− 0.179 to 0.106)	235	− 0.072 (− 0.219 to 0.074)

The data are standardised regression coefficients and their 95% confidence intervals adjusted for age, sex, and parental education at 8-year follow-up. The data on the associations between change in physical fitness and cognitive functions were also adjusted for physical fitness score at baseline. Statistically significant associations are bolded

*kPa* kilopascal, *LBM* lean body mass, *VO*_*2peak*_ peak oxygen uptake, *W*_*max*_ maximal workload

### Associations of Average Physical Fitness and Changes in Physical Fitness Over 8 Years with Mental Health at 8-Year-Follow-Up

A higher average motor fitness was associated with lower perceived stress and depressive symptoms at the 8-year follow-up after adjustment for age, sex, and parental education (Table 2). The inverse association between average motor fitness and depressive symptoms was attenuated after further adjustment for screen time at 8-year follow-up (*β* =  − 0.142, 95% CI − 0.007 to 0.291, *p* = 0.062).

Higher levels of average cardiorespiratory fitness (*W*_max_/kg of LBM and *V*O_2peak_/kg of LBM) were associated with lower perceived stress and depressive symptoms at the 8-year follow-up. Further adjustment for screen time attenuated the association between average *W*_max_/kg of LBM and perceived stress (*β* =  − 0.118, 95% CI − 0.250 to 0.015, *p* = 0.081). The inverse association of average *V*O_2peak_/kg of LBM with perceived stress was attenuated after additional adjustment for physical activity (β =  − 0.120, 95% CI − 0.269 to 0.031, *p* = 0.120) or screen time (β =  − 0.115, 95% CI − 0.264 to 0.034, *p* = 0.129).

A larger increase in cardiorespiratory fitness (*W*_max_/kg of LBM and *V*O_2peak_/kg of LBM) over the follow-up was associated with lower perceived stress and depressive symptoms at the 8-year follow-up after adjustment for age, sex, parental education, and corresponding measure of cardiorespiratory fitness at the first assessment point (Table 2). Further adjustment for screen time at 8-year follow-up attenuated the association of change in *W*_max_/kg of LBM with perceived stress (*β* =  − 0.094, 95% CI − 0.252 to 0.064, *p* = 0.244) and depressive symptoms (*β* =  − 0.139, 95% CI − 0.297 to 0.018, *p* = 0.083).

### Sex as a Modifier of the Associations of Physical Fitness with Cognition and Mental Health

The change in muscular fitness assessed by the standing long jump test over 8 years was inversely associated with depressive symptoms in girls (*β* =  − 0.256, 95% CI − 0.457 to − 0.056, *p* = 0.013), but not in boys (*β* =  − 0.061, 95% CI − 0.247 to 0.125, *p* = 0.515, *p* = 0.032 for interaction). Further adjustment for screen time at the 8-year follow-up attenuated the inverse association between change in standing long jump performance and depressive symptoms in girls (*β* =  − 0.178, 95% CI − 0.380 to 0.025, *p* = 0.086). The modifying effect of sex on the associations of other measures of physical fitness over 8 years with measures of cognition and mental health at the 8-year follow-up are presented in the ESM (Online Resource S2).

## Discussion

In our longitudinal study, we found that higher average motor fitness, assessed by the 10 × 5-m shuttle run test in childhood and adolescence, was associated with better cognition in adolescence. Furthermore, higher cardiorespiratory fitness across the follow-up was associated with lower levels of perceived stress and depressive symptoms in adolescence. Intriguingly, while our findings suggest that average motor fitness in childhood and adolescence, rather than changes in motor fitness across the follow-up, was directly related to cognition in adolescence; better average but also improvement in cardiorespiratory fitness over the follow-up was inversely associated with perceived stress and depressive symptoms in adolescence. Consequently, our results highlight the multifaceted relationships between physical fitness, cognition, and mental health.

Previous cross-sectional and short-term longitudinal studies have suggested inconclusive evidence for an association between motor fitness and cognition in children and adolescents [16, 22, 51]. The results of our study, spanning over 8 years, suggest that sustained higher motor fitness from childhood to adolescence is associated with better cognition in adolescence. However, the change in motor fitness was not related to cognition. Therefore, these findings suggest that a cumulative impact of motor fitness may have a pronounced influence on cognitive development. This association may be due to the parallel development of motor fitness and brain structures, such as the cerebellum and some cortical areas, contributing to the association between sustained motor fitness and cognitive functions [42, 52]. Moreover, the weak association between the change in motor fitness and cognition could be attributed to the variability in individual developmental trajectories. Participants might have experienced fluctuations in motor fitness over the 8 years, but these short-term changes may not have translated into meaningful cognitive changes. Therefore, it is plausible that cognitive benefits are more closely tied to the overall level of motor fitness than to specific fluctuations within the study period.

In contrast to the results of previous studies [18], we found that cardiorespiratory fitness was not associated with cognition. Our approach to normalise measures of cardiorespiratory fitness for LBM reduces the potential confounding role of body adiposity. The associations previously observed between cardiorespiratory fitness and cognition may have been influenced by body adiposity, which is strongly associated with cognition [53] and often intertwines with common measures of cardiorespiratory fitness metrics, such as the 20-m shuttle run test performance [54]. As such, we have previously observed that cardiorespiratory fitness assessed by the 20-m shuttle run test exhibits stronger associations with cognition than *V*O_2peak_/kg of LBM in children with overweight/obesity [55]. Aligned with our findings, body adiposity was a stronger determinant of brain metabolism than cardiorespiratory fitness normalised for LBM in adults [56]. Nevertheless, cardiorespiratory fitness normalised for LBM may be more strongly associated with cognition assessed after a bout of exercise than that assessed after a rest, suggesting a complicated interaction between cardiorespiratory fitness and physical activity [57]. Finally, in line with previous studies [58, 59], we observed no associations between muscular fitness and global cognition score, suggesting that muscular fitness may not be the primary component of physical fitness related to cognition during childhood and adolescence. Nevertheless, we found that better lower limb muscular fitness was associated with faster reaction times in the psychomotor function and attention tasks, suggesting that muscular fitness might be related to some specific components of cognition. These positive associations between muscular fitness and cognition may reflect participation in muscle-strengthening physical activities that have been shown to increase circulating neurotrophic factors and enhance brain haemodynamic responses [60].

In line with cross-sectional and a few longitudinal studies [17], we found that average cardiorespiratory fitness in childhood and adolescence was directly associated with mental health. Importantly, we observed that a larger improvement in cardiorespiratory fitness from childhood to adolescence was associated with lower levels of perceived stress and depressive symptoms in adolescence. In addition, we found that better average motor fitness in childhood and adolescence was associated with better mental health in adolescence, supporting the results from cross-sectional studies [17]. These observations advocate for the investment in physical fitness from early life as a potential strategy for mitigating mental health issues in adolescence. The underlying mechanisms may involve the enhancement of self-esteem, self-concept, self-efficacy, and overall mental and physical resilience through higher motor fitness and improved cardiorespiratory fitness [61, 62]. However, our results suggest that while cardiorespiratory fitness may be independently associated with mental health, screen time may moderate some associations between cardiorespiratory fitness and mental health. The explanation for this finding may be that adolescents with lower levels of cardiorespiratory fitness also have higher levels of screen time that has been inversely associated with mental health in children and adolescents [61–63]. Therefore, comprehensive lifestyle interventions that focus on reducing sedentary time and improving cardiorespiratory fitness may provide meaningful benefits for mental health in youth.

While most associations were similar in girls and boys, we observed that improving standing jump over 8 years was associated with fewer depressive symptoms in girls, but the corresponding association in boys was weak and not statistically significant. Therefore, it is possible that while higher levels of sustained cardiorespiratory fitness and improved cardiorespiratory fitness benefit both girls’ and boys’ mental health, improvements in lower limb power may be particularly important for girls. While the mechanism of sex-specific associations between physical fitness and mental health could be multifaceted, lower limb power is important for performance in a range of sports and physical activities (e.g., dance, gymnastics, ball games, and track and field). Therefore, improvement in this fitness component may contribute to enhanced self-esteem, self-concept, and body image, which may lead to reductions in depression [61, 63]. However, our results also suggest that the mental health of girls with better standing long jump performance may benefit from their lower levels of screen time.

The strengths of the present study include a long-term follow-up from childhood to adolescence, and well-characterised measures of physical fitness, cognition, and mental health. We also had an opportunity to control the data for several confounding factors. However, our study sample included relatively healthy (both physically and mentally) children and adolescents from a general population. Therefore, our findings may not be generalised to populations including children and adolescents with higher levels of overweight and obesity or clinical cognitive or mental disorders. We adjusted the data on the associations of cardiorespiratory, motor, and muscular fitness in childhood and adolescence with the measures of cognition in adolescence by the Raven’s score in childhood. However, using the same cognition measure as in adolescence for adjustment would have been optimal. As cognition is a broad concept that can be assessed using various tests, further replication studies are also required to confirm our findings. Finally, a major weakness of our study was that we did not have data on mental health at baseline, and therefore, we cannot rule out that better mental health in childhood has contributed to improved cardiorespiratory fitness and later mental health.

## Conclusion

We found that higher average motor fitness in childhood and adolescence was associated with better cognition in adolescence, whereas higher average cardiorespiratory fitness and larger improvement in cardiorespiratory fitness in childhood and adolescence predicted better mental health in adolescence. Our findings highlight the importance of assessing several indices of physical fitness to quantify its role in cognition and mental health in a research among children and adolescents. These results also suggest that promoting a variety of physical activities and reducing recreational screen time, thereby improving physical fitness, should be used as one option to improve cognition and mental health in youth. Future studies are warranted to investigate the mechanisms underlying the relationship between physical fitness and cognitive and mental well-being.

## Supplementary Information

Below is the link to the electronic supplementary material.

Supplementary file1 (DOCX 51 KB)

## Declarations

### Data transparency

The data are not publicly available due to research ethical reasons and because the owner of the data is the University of Eastern Finland and not the research group. The pseudonymised data are available upon reasonable request from the corresponding author.
