# Supplementary material for: Childhood Physical Fitness as a Predictor of Cognition and Mental Health in Adolescence: The PANIC Study
Source: Sports Med. 2024 Sep 10;55(2):487–97. doi: 10.1007/s40279-024-02107-z (PMC11947037; doi:10.1007/s40279-024-02107-z)
Supplement: Supplementary file 1 — Supplementary file1 (DOCX 51 KB) [file 40279_2024_2107_MOESM1_ESM.docx]

**Online Resource - Sports Medicine**

Childhood physical fitness as a predictor of cognition and mental health in adolescence – The PANIC study

Eero A. Haapala^1,2^, Marja H. Leppänen^1^, Hannamari Skog^2^, David R. Lubans^3,4^, Anna Viitasalo^2^, Niina Lintu^2^, Petri Jalanko^1,5^, Sara Määttä^6^, Timo A. Lakka^2,7,8^

^1^Faculty of Sport and Health Sciences, University of Jyväskylä, Jyväskylä, Finland; ^2^Institute of Biomedicine, School of Medicine, University of Eastern Finland, Kuopio, Finland; ^3^Centre for Active Living and Learning, College of Human and Social Futures, University of Newcastle, Callaghan, New South Wales, Australia; ^4^Hunter Medical Research Institute, New Lambton Heights, New South Wales, Australia; ^5^Helsinki Clinic for Sports and Exercise Medicine, Foundation for Sports and Exercise Medicine, Helsinki, Finland; ^6^Department of Clinical Neurophysiology, Kuopio University Hospital, Kuopio, Finland; ^7^Department of Clinical Physiology and Nuclear Medicine, Kuopio University Hospital, Kuopio, Finland; ^8^Foundation for Research in Health Exercise and Nutrition, Kuopio Research Institute of Exercise Medicine, Kuopio, Finland.

**Correspondence:** Dr. Eero A. Haapala, PhD, Sports & Exercise Medicine, Faculty of Sport and Health Sciences, University of Jyväskylä, Jyväskylä, Finland, tel. +358408054210, [eero.a.haapala@jyu.fi](mailto:eero.a.haapala@jyu.fi)

**Online Resource 1.**

**1 METHODS**

**1.1 Assessment of cognitive functions**

Non-verbal reasoning skills were assessed by a paper-pencil version of the Raven’s Standard Progressive Matrices (Raven’s SPM) [1]. The Raven’s SPM includes five sets of 12 items. Each test page includes a large item or a pattern of items and six small items. The participant was asked to select the correct small item, which completes the large item or the set of items. The test score was the number of correct answers, ranging from zero to 60. The Raven’s SPM requires the ability to find similarities, differences, and discrete patterns and does not depend on acquired knowledge or language skills. The Raven’s SPM has been suggested to represent all-core components of executive functions [2], and it is a well-validated and reproducible measure of cognition in children, adolescents, and adults [3–5].

Paired-associate learning, psychomotor function, attention, and working memory were assessed by a computerized CogState cognitive test battery (CogState Ltd, Melbourne, Australia) using a desktop personal computer [6]. The construct validity of the CogState test battery has been demonstrated in a large group of healthy children and adults [7,8]. The test can be used in different cultures since there are only minimal language requirements to undertake the test battery.

Paired-associate learning was assessed by the continuous paired associate learning task. The test had two parts. First, the participants were asked to learn and remember abstract pictures and hidden patterns in different locations. Second, the participants were requested to recall where the re-displayed hidden picture had been located. The test score was the errors during all performance sequences. A lower score indicated better performance.

The psychomotor function was assessed with the detection task. The participants were asked to click a button as quickly as possible when a playing card flipped over on a computer screen during the test. The test score was based on the speed of performance using mean of the log10 transformed reaction times for correct responses. A lower score indicated better performance.

Attention was assessed by the identification task. The participants were asked to choose whether a revealed card was red or not during the task. The participants were required to press "yes" for the red card, and “no” for the black card. The test score was based on the speed of performance using mean of the log10 transformed reaction times for correct responses. A lower score implied better performance.

The reaction time in the working memory test was assessed by the one-back task. The participants were asked, using buttons “yes” or “no”, to answer whether the present card was the same as the previous card. The test score was based on the speed of performance using mean of the log10 transformed reaction times for correct responses. A lower score implied better performance.

Working memory was assessed by the two-back task. The participants were asked (using buttons “yes” or “no”) to answer whether a present card was the same as the card presented two cards ago. The test score was based on the response accuracy using the arcsine transformation of the square root of the proportion of correct responses. A higher score indicated better performance.

**Online Resource 2**

**2 RESULTS**

**2.1 Associations of physical fitness at baseline, 2-year follow-up, and 8-year follow-up with individual measures of cognition at 8-year follow-up**

*2.1.1 Physical fitness assessed at baseline*

A higher motor fitness at baseline was associated with a better global cognition score, faster reaction time in the one-back task, and a better response accuracy at two-back task at the 8-year follow-up after adjustment for age, sex, and parental education (Supplementary Tables S1 and S2). A higher muscular fitness (standing long jump performance) was associated with faster reaction times in the detection and identification tasks. A direct association between motor fitness and two-back task accuracy attenuated after further adjustment for Raven’s CPM score at baseline (β=-0.119, 95% confidence intervals [CI]=-0.242 to 0.005, p=0.059). Further adjustments for pubertal status, body fat percentage, physical activity, or screen time at 8-year follow-up, or Raven’s Coloured Progressive Matrices (CPM) score at baseline had no effect on the magnitude of other associations.

A higher muscular fitness (standing long jump performance) at baseline was associated with a higher global cognition score (Girls: β=0.315, 95% CI=0.137 to 0.493, p<0.001; Boys: 0.055, 95% CI=-0.127 to 0.236, p=0.553, p=0.014 for interaction) and faster reaction times in the detection (Girls: β=-0.254, 95% CI=-0.429 to -0.078, p=0.005; Boys: β=-0.126, 95% CI=-0.307 to 0.054, p=0.169, p=0.034 for interaction) and the one-back tasks (Girls: β=-0.225, -0.415 to -0.036, p=0.020; Boys: β=-0.016, -0.198 to 0.167, p=0.863, p=0.084 for interaction) at the 8-year follow-up in girls but not in boys. Further adjustments had no effect on the magnitude of these associations.

*2.1.2 Physical fitness assessed at the 2-year follow-up*

A higher motor fitness at the 2-year follow-up was associated with a faster reaction time in the one-back task at the 8-year follow-up after adjustment for age, sex, and parental education (Supplementary Table S2). A higher muscular fitness (standing long jump performance) was associated with a higher global cognition score and faster reaction times in the detection and identification tasks (Supplementary Tables S1 and S2). Further adjustments for pubertal status, body fat percentage, physical activity, or screen time at the 8-year follow-up or Raven’s CPM score at a 2-year follow-up had no effect on the magnitude of other associations.

*2.1.3 Physical fitness assessed at the 8-year follow-up*

A higher motor fitness at the 8-year follow-up was associated with a higher global cognition score, faster reaction times in the detection and the identification tasks, and better accuracy in the two-back task at the 8-year follow-up (Supplementary Tables S1 and S2). The direct association between motor fitness and two-back task accuracy attenuated after further adjustment for body fat percentage at the 8-year follow-up (β=-0.146, 95% CI=-0.292 to 0.027, p=0.101). A higher muscular fitness (standing long jump performance) at the 8-year follow-up was associated with faster reaction times in the detection and identification tasks at the 8-year follow-up. Further adjustments for pubertal status, body fat percentage, physical activity, or screen time at 8-year follow-up had no effect on the magnitude of other associations.

**2.2 Associations of average physical fitness over eight years with individual measures of cognition at 8-year follow-up**

A higher average motor fitness was associated with a faster reaction time in the detection task after adjustment for age, sex, and parental education (Supplementary Table S3). A higher average muscular fitness (standing long jump performance) was associated with faster reaction times in the detection and identification tasks. Further adjustments for pubertal status, body fat percentage, physical activity, or screen time at 8-year follow-up or Raven’s CPM score at baseline had no effect on the magnitude of these associations.

**2.3 Associations of changes in physical fitness over eight years with individual measures of cognition at 8-year follow-up**

A larger improvement in motor fitness, as indicated by larger reduction in the 10 x 5m shuttle run test time over eight years, was associated with lower response time in the detection task after adjustment for age, sex, parental education, and motor fitness at baseline (Supplementary Table S3). Moreover, a larger improvement in muscular fitness, as indicated by a larger improvement in the standing long jump performance, was associated with a faster response time in the detection task in boys (β=-0.207, 95% CI=-0.389 to -0.025, p=0.026) but not in girls (β=0.030, 95% CI=-0.158 to 0.218, p=0.736, p=0.044 for interaction). Further adjustments had no effect on these associations.

**2.4 Associations of physical fitness at baseline, 2-year follow-up and 8-year follow-up with mental health at 8-year-follow-up**

*2.4.1 Physical fitness assessed at baseline*

A higher cardiorespiratory fitness (maximal workload normalised for lean body mass [W_max_/kg of LBM]) at baseline was associated with lower depressive symptoms at the 8-year follow-up after adjustment for age, sex, and parental education (Supplementary Table S1). Further adjustments had no effect on this association.

A higher muscular fitness (standing long jump performance) at baseline was associated with higher perceived depressive symptoms in girls (β=0.221, 95% CI=0.027 to 0.415, p=0.026) but not in boys (β=-0.098, 95% CI=-0.280 to 0.084, p=0.290, p=0.024 for interaction).

*2.4.2 Physical fitness assessed at 2-year follow-up*

Higher cardiorespiratory fitness (W_max_/kg of LBM) and better muscular fitness (standing long jump performance) at 2-year follow-up were associated with lower depressive symptoms at the 8-year follow-up after adjustment for age, sex, and parental education (Supplementary Table S1). Further adjustment for screen time at 8-year follow-up attenuated the inverse association of cardiorespiratory fitness (W_max_/kg of LBM) with depressive symptoms (β=-0.117, 95% CI=-0.239 to 0.006, p=0.061). Other adjustments had no effect on these associations.

Lower motor fitness at 2-year follow-up was associated with higher perceived stress at the 8-year follow-up in boys (β=0.247, 95% CI=-0.060 to 0.434, p=0.010), but not in girls (β=-0.013, 95% CI=-0.213 to 0.187, p=0.895, p=0.052 for interaction). Similarly, lower muscular fitness (standing long jump performance) at 2-year follow-up was associated with higher perceived stress in boys (β=-0.266, 95% CI=-0.444 to -0.087, p=0.004), but not in girls (β=0.126, 95% CI=-0.072 to 0.325, p=0.210, p=0.004 for interaction). Further adjustments had no effect on these associations. Muscular fitness (standing long jump performance) at 2-year follow-up had a weak positive association with perceived depressive symptoms at 8-year follow-up in girls (β=0.048, 95% CI=-0.157 to 0.253, p=0.644) but had an inverse association in boys (β=-0.322, 95% CI=-0.498 to -0.146, p<0.001, p=0.021 for interaction). Further adjustments had no effect on these associations.

*2.4.3 Physical fitness assessed at 8-year follow-up*

A higher cardiorespiratory fitness (W_max_/kg of LBM and VO_2peak_/kg of LBM), motor fitness, and muscular fitness (standing long jump performance) at the 8-year follow-up were associated with lower perceived stress and depressive symptoms at the 8-year follow-up (Supplementary Table S1). The inverse association between motor fitness and depressive symptoms attenuated after additional adjustment for physical activity (β=0.138, 95% CI=-0.005 to 0.281, p=0.059) and screen time (β=0.059, 95% CI=-0.082 to 0.200, p=0.420) at the 8-year follow-up. Further adjustment for screen time at 8-year follow-up weakened the association between muscular fitness (standing long jump) and depressive symptoms (β=0.154, 95% CI=-0.112 to 0.042, p=0.154).

**REFERENCES**

1. Abdel-Khalek A. Reliability and Factorial Validity of the Standard Progressive Matrices among Kuwaiti Children Ages 8 to 15 Years. Percept Mot Skills. 2005;101:409–12.

2. Diamond A. Executive Functions. Ann Rev Psychol. 2013;64:135–68.

3. Cotton SM, Kiely PM, Crewther DP, Thomson B, Laycock R, Crewther SG. A normative and reliability study for the Raven’s Coloured Progressive Matrices for primary school aged children from Victoria, Australia. Person Individ Diff. 2005;39:647–59.

4. Raven J. Raven Progressive Matrices. In: McCallum RS, editor. Handbook of Nonverbal Assessment. Boston, MA: Springer US; 2003. p. 223–37.

5. Raven J. The Raven’s Progressive Matrices: Change and Stability over Culture and Time. Cognitive Psychology. 2000;41:1–48.

6. Skog H, Lintu N, Haapala HL, Haapala EA. Associations of cardiorespiratory fitness, adiposity, and arterial stiffness with cognition in youth. Physiol Reps. 2020;8:e14586.

7. Maruff P, Thomas E, Cysique L, Brew B, Collie A, Snyder P, et al. Validity of the CogState Brief Battery: Relationship to Standardized Tests and Sensitivity to Cognitive Impairment in Mild Traumatic Brain Injury, Schizophrenia, and AIDS Dementia Complex. Arch Clin Neuropsychol. 2009;24:165–78.

8. Mollica CM, Maruff P, Collie A, Vance A. Repeated Assessment of Cognition in Children and the Measurement of Performance Change. Child Neuropsychol. 2005;11:303–10.

| Online Resource Table S1. Associations of physical fitness at baseline, 2-year follow-up, and 8-year follow-up with cognition and mental health at 8-year follow-up. | | | | | | | |
| --- | --- | --- | --- | --- | --- | --- | --- |
|  | N | Global cognition score | N | Perceived stress score | N | Depressive symptoms score |  |
| **Baseline** |  |  |  |  |  |  |  |
| Cardiorespiratory fitness (Wmax/kg of LBM) | 250 | 0.056 (-0.072 to 0.185) | 252 | -0.076 (-0.197 to 0.046) | 244 | **-0.212 (-0.336 to -0.088)** |  |
| Motor fitness (10 x 5m shuttle run test, s) | 241 | **-0.179 (-0.307 to -0.051)** | 243 | 0.121 (-0.003 to 0.245) | 236 | 0.120 (-0.009 to 0.248) |  |
| Muscular fitness (Standing long jump, cm) | 243 | **0.184 (0.053 to 0.314)** | 245 | -0.034 (-0.161 to 0.093) | 237 | 0.063 (-0.069 to 0.195) |  |
| Muscular fitness (Handgrip strength, kPa/LBM) | 262 | -0.046 (-0.167 to 0.076) | 262 | -0.048 (-0.163 to 0.068) | 254 | -0.050 (-0.171 to 0.071) |  |
| **2-year follow-up** |  |  |  |  |  |  |  |
| Cardiorespiratory fitness (Wmax/kg of LBM) | 253 | 0.012 (-0.114 to 0.139) | 254 | -0.076 (-0.196 to 0.045) | 246 | **-0.161 (-0.286 to -0.038)** |  |
| Cardiorespiratory fitness (VO_2peak_ / kg of LBM) | 221 | -0.035 (-0.171 to 0.100) | 222 | 0.000 (-0.130 to 0.131) | 215 | -0.109 (-0.244 to 0.026) |  |
| Motor fitness (10 x 5m shuttle run test, s) | 224 | -0.090 (-0.228 to 0.047) | 226 | 0.111 (-0.021 to 0.242) | 219 | 0.104 (-0.033 to 0.240) |  |
| Muscular fitness (Standing long jump, cm) | 227 | **0.164 (0.025 to 0.303)** | 229 | -0.095 (-0.229 to 0.040) | 222 | **-0.144 (-0.282 to -0.006)** |  |
| Muscular fitness (Handgrip strength, kPa/kg of LBM) | 254 | -0.039 (-0.163 to 0.084) | 256 | -0.024 (-0.142 to 0.094) | 248 | -0.018 (-0.141 to 0.106) |  |
| **8-year follow-up** |  |  |  |  |  |  |  |
| Cardiorespiratory fitness (Wmax/kg of LBM) | 237 | 0.101 (-0.028 to 0.23) | 239 | **-0.157 (-0.281 to -0.033)** | 237 | **-0.226 (-0.351 to -0.102)** |  |
| Cardiorespiratory fitness (VO_2peak_ / kg of LBM) | 213 | 0.07 (-0.069 to 0.208) | 215 | **-0.195 (-0.327 to -0.064)** | 214 | **-0.267 (-0.399 to -0.135)** |  |
| Motor fitness (10 x 5m shuttle run test, s) | 241 | **-0.208 (-0.346 to -0.07)** | 243 | **0.151 (0.016 to 0.287)** | 241 | **0.153 (0.013 to 0.292)** |  |
| Muscular fitness (Standing long jump, cm) | 240 | 0.130 (-0.026 to 0.286) | 242 | **-0.173 (-0.324 to -0.023)** | 240 | **-0.200 (-0.244 to -0.046)** |  |
| Muscular fitness (Handgrip strength, kPa/kg of LBM) | 239 | -0.065 (0.200 to 0.068) | 241 | -0.046 (-0.174 to 0.082) | 239 | -0.072 (-0.204 to 0.059) |  |

The data are standardised regression coefficients and their 95% confidence intervals adjusted for age, sex, and parental education at 8-year follow-up. Statistically significant associations are bolded. kPa, kilopascals; LBM, lean body mass; VO_2peak_, peak oxygen uptake; Wmax, maximal power output.

| Online Resource Table S2. Associations of physical fitness at baseline, 2-year follow-up, and 8-year follow-up with individual measures of cognition at 8-year follow-up. | | | | | | | |
| --- | --- | --- | --- | --- | --- | --- | --- |
|  | N | Raven’s SPM Score | CPAL (errors) | DET (reaction time) | IDN (reaction time) | ONB (reaction time) | TWOB (accuracy) |
| **Baseline** |  |  |  |  |  |  |  |
| Cardiorespiratory fitness (Wmax/kg of LBM) | 250–252 | 0.017 (0.111 to 0.144) | 0.011 (-0.114 to 0.136) | -0.06 (-0.186 to 0.065) | -0.039 (-0.168 to 0.091) | -0.027 (-0.154 to 0.101) | 0.069 (-0.060 to 0.198) |
| Motor fitness (10 x 5m shuttle run test, s) | 241–243 | -0.066 (-0.195 to 0.062) | 0.045 (-0.082 to 0.173) | 0.107 (-0.020 to 0.234) | 0.117 (-0.014 to 0.247) | **0.162 (0.035 to 0.289)** | **-0.149 (-0.279 to -0.019)** |
| Muscular fitness (Standing long jump, cm) | 243–245 | 0.012 (-0.120 to 0.143) | -0.007 (-0.137 to 0.124) | **-0.196 (-0.325 to -0.068)** | **-0.207 (-0.338 to -0.075)** | -0.123 (-0.254 to 0.008) | 0.120 (-0.012 to 0.253) |
| Muscular fitness (Handgrip strength, kPa/kg of LBM) | 262–264 | -0.016 (-0.137 to 0.105) | 0.046 (-0.072 to 0.165) | 0.034 (-0.086 to 0.154) | 0.002 (-0.121 to 0.125) | 0.034 (-0.087 to 0.155) | -0.027 (-0.149 to 0.096) |
| **2-year follow-up** |  |  |  |  |  |  |  |
| Cardiorespiratory fitness (Wmax/kg of LBM) | 253–254 | 0.011 (-0.115 to 0.137) | 0.057 (-0.067 to 0.180) | -0.022 (-0.147 to 0.103) | -0.062 (-0.189 to 0.066) | 0.042 (-0.173 to 0.075) | 0.048 (-0.079 to 0.175) |
| Cardiorespiratory fitness (VO_2peak_/kg of LBM) | 221–222 | 0.014 (-0.121 to 0.149) | 0.032 (-0.102 to 0.165) | -0.022 (-0.156 to 0.111) | -0.006 (-0.143 to 0.132) | 0.105 (-0.028 to 0.238) | -0.029 (-0.043 to 0.272) |
| Motor fitness (10 x 5m shuttle run test, s) | 224–226 | 0.012 (-0.124 to 0.149) | -0.007 (-0.143 to 0.129) | 0.052 (-0.083 to 0.188) | 0.098 (-0.041 to 0.236) | **0.146 (0.011 to 0.281)** | -0.057 (-0.196 to 0.083) |
| Muscular fitness (Standing long jump, cm) | 227–229 | 0.054 (-0.085 to 0.193) | -0.027 (-0.165 to 0.111) | **-0.159 (-0.295 to -0.023)** | **-0.191 (-0.330 to -0.052)** | -0.132 (-0.269 to 0.005) | 0.041 (-0.101 to 0.183) |
| Muscular fitness (Handgrip strength, kPa/kg of LBM) | 254–256 | 0.026 (-0.097 to 0.149) | -0.015 (-0.135 to 0.106) | 0.028 (-0.094 to 0.150) | 0.039 (-0.086 to 0.164) | 0.076 (-0.046 to 0.197) | -0.029 (-0.154 to 0.095) |
| **8-year follow-up** |  |  |  |  |  |  |  |
| Cardiorespiratory fitness (Wmax/kg of LBM) | 237–239 | 0.049 (-0.08 to 0.178) | -0.014 (-0.141 to 0.112) | -0.120 (-0.247 to 0.007) | -0.077 (-0.208 to 0.054) | -0.014 (-0.144 to 0.116) | 0.088 (-0.043 to 0.219) |
| Cardiorespiratory fitness (VO_2peak_/kg of LBM) | 213–215 | 0.036 (-0.100 to 0.172) | -0.022 (-0.156 to 0.112) | -0.051 (-0.188 to 0.086) | -0.091 (-0.231 to 0.049) | -0.003 (-0.136 to 0.142) | 0.052 (-0.089 to 0.191) |
| Motor fitness (10 x 5m shuttle run test, s) | 241–243 | -0.13 (-0.270 to 0.010) | -0.006 (-0.143 to 0.131) | **0.231 (0.096 to 0.366)** | **0.145 (0.004 to 0.287)** | 0.076 (-0.064 to 0.217) | **-0.165 (-0.306 to -0.024)** |
| Muscular fitness (Standing long jump, cm) | 240–242 | -0.012 (-0.168 to 0.144) | 0.036 (-0.116 to 0.188) | **-0.179 (-0.331 to -0.027)** | **-0.165 (-0.322 to -0.008)** | -0.05 (-0.206 to 0.107) | 0.13 (-0.028 to 0.288) |
| Muscular fitness (Handgrip strength, kPa/kg of LBM) | 239–241 | 0.011 (-0.122 to 0.144) | 0.076 (-0.052 to 0.205) | 0.027 (-0.104 to 0.158) | 0.071 (-0.064 to 0.206) | -0.053 (-0.186 to 0.080) | -0.128 (-0.262 to 0.01) |

The data are standardised regression coefficients and their 95% confidence intervals adjusted for age, sex, and parental education at 8-year follow-up. Statistically significant associations are bolded. CPAL, continuous associated learning; DET, detection task; IDN, identification task; kPa, kilopascals; LBM, lean body mass; ONB, one back task; Raven’s SPM, Raven’s Standard Progressive Matrices; TWOB, two-back task; VO_2peak_, peak oxygen uptake; Wmax, maximal power output.

| Online Resource Table S3. Associations of average physical fitness and changes in physical fitness over eight years with individual measures of cognition at 8-year follow-up. | | | | | | | |
| --- | --- | --- | --- | --- | --- | --- | --- |
|  | N | Raven’s CPM Score | CPAL (errors) | DET (reaction time) | IDN (reaction time) | ONB (reaction time) | TWOB (accuracy) |
| **Average physical fitness** |  |  |  |  |  |  |  |
| Cardiorespiratory fitness (Wmax/kg of LBM) | 217–218 | -0.021 (-0.158 to 0.115) | 0.028 (-0.105 to 0.161) | -0.081 (-0.214 to 0.053) | -0.082 (-0.220 to 0.056) | 0.009 (-0.128 to 0.145) | 0.057 (-0.081 to 0.195) |
| Cardiorespiratory fitness (VO_2peak_ / kg of LBM) | 183–184 | 0.006 (-0.143 to 0.155) | -0.020 (-0.169 to 0.128) | -0.077 (-0.226 to 0.072) | -0.097 (-0.251 to 0.056) | 0.025 (-0.125 to 0.176) | 0.003 (-0.151 to 0.157) |
| Motor fitness (10 x 5m shuttle run test, s) | 189–191 | -0.079 (-0.233 to 0.075) | 0.004 (-0.150 to 0.159) | **0.165 (0.014 to 0.317)** | 0.113 (-0.044 to 0.270) | 0.130 (-0.023 to 0.283) | -0.103 (-0.261 to 0.055) |
| Muscular fitness (Standing long jump, cm) | 191–193 | -0.030 (-0.198 to 0.139) | 0.077 (-0.091 to 0.245) | **-0.213 (-0.375 to -0.050)** | **-0.198 (-0.366 to -0.029)** | -0.047 (-0.216 to 0.122) | 0.002 (-0.171 to 0.175) |
| Muscular fitness (Handgrip strength, kPa/kg of LBM) | 227–229 | 0.017 (-0.115 to 0.149) | 0.043 (-0.086 to 0.171) | 0.009 (-0.122 to 0.139) | 0.021 (-0.113 to 0.155) | 0.003 (-0.129 to 0.135) | -0.071 (-0.205 to 0.063) |
| **Change in physical fitness** |  |  |  |  |  |  |  |
| Cardiorespiratory fitness (Wmax/kg of LBM) | 224-226 | 0.03 (-0.133 to 0.193) | -0.033 (-0.193 to 0.127) | -0.114 (-0.273 to 0.045) | -0.056 (-0.221 to 0.109) | 0.021 (-0.143 to 0.185) | 0.072 (-0.093 to 0.238) |
| Cardiorespiratory fitness (VO_2peak_ / kg of LBM) | 183-184 | 0.043 (-0.138 to 0.223) | -0.074 (-0.253 to 0.106) | -0.014 (-0.194 to 0.166) | -0.145 (-0.33 to 0.041) | -0.036 (-0.218 to 0.146) | 0.088 (-0.098 to 0.273) |
| Motor fitness (10 x 5m shuttle run test, s) | 222-224 | -0.095 (-0.28 to 0.09) | -0.003 (-0.185 to 0.179) | **0.229 (0.049 to 0.410)** | 0.141 (-0.046 to 0.328) | -0.032 (-0.216 to 0.153) | -0.109 (-0.295 to 0.077) |
| Muscular fitness (Standing long jump, cm) | 222-223 | -0.053 (-0.215 to 0.11) | 0.063 (-0.097 to 0.224) | -0.091 (-0.248 to 0.067) | -0.036 (-0.200 to 0.127) | 0.044 (-0.120 to 0.207) | -0.037 (-0.203 to 0.128) |
| Muscular fitness (Handgrip strength, kPa/kg of LBM) | 235-237 | 0.011 (-0.138 to 0.159) | 0.064 (-0.079 to 0.207) | 0.019 (-0.127 to 0.164) | 0.095 (-0.055 to 0.245) | -0.06 (-0.208 to 0.089) | -0.144 (-0.294 to 0.005) |

The data are standardised regression coefficients and their 95% confidence intervals adjusted for age, sex, and parental education at 8-year follow-up. The data one the associations between change in physical fitness and cognitive functions were also adjusted for physical fitness score at baseline. Statistically significant associations are bolded. CPAL, continuous associated learning; DET, detection task; IDN, identification task; kPa, kilopascals; LBM, lean body mass; ONB, one back task; Raven’s SPM, Raven’s Standard Progressive Matrices; TWOB, two-back task; VO_2peak_, peak oxygen uptake; Wmax, maximal power output.
